# Preventive Effects of the Chinese Herbal Medicine Prescription Tangkuei Decoction for Frigid Extremities on Sciatic Neuropathy in Streptozotocin-Induced Diabetic Rats

**DOI:** 10.1155/2016/9138328

**Published:** 2016-02-29

**Authors:** Pengsong Liu, Yuanyuan Bian, Hong Zhang, Aiming Jia

**Affiliations:** Department of Traditional Chinese Medicine, Second Affiliated Hospital of Dalian Medical University, Dalian, Liaoning 116023, China

## Abstract

Ischemia and hypoxia are important physiological changes in diabetic peripheral neuropathy (DPN). Chinese herbal medicine prescription Tangkuei Decoction for Frigid Extremities (TDFE) is useful for increasing blood flow. To help determine whether TDFE could protect the peripheral nerves of diabetic patients from the degeneration caused by high blood glucose, TDFE was administered to streptozotocin-induced diabetic rats for 6 or 12 weeks. Plantar thermal stimulation reaction time thresholds, sciatic nerve conduction velocities, and the levels of HIF-1*α* mRNA, HIF-1*α* protein, VEGF protein, and the endothelial marker vWF in sciatic nerves were measured at the end of the sixth and twelfth weeks. The thermal thresholds and sciatic nerve conduction velocities of the rats differed after 12 weeks, and the sciatic nerves of the diabetic rats that were given TDFE displayed higher levels of HIF-1*α* protein, VEGF protein, and HIF-1*α* mRNA than those of the diabetic model rats. The results at 6 weeks differed from those at 12 weeks. These results suggest that the early preventive application of TDFE effectively delayed the development of DPN and that TDFE increased HIF-1*α* mRNA levels in the sciatic nerves of diabetic rats through 12 weeks of treatment.

## 1. Introduction

Diabetes mellitus (DM) is prevalent worldwide, and neuropathy is its most disabling and serious complication [1]. Resulting in painful and insensitive extremities and neuropathic ulceration and amputation, diabetic peripheral neuropathy (DPN) is one of the most common complications of both type 1 and type 2 diabetes [2]. Diabetes is associated with the failure of various organs, especially the blood vessels [3], and ischemia and hypoxia are considered important contributing factors in the development of DPN [2, 4]. The role of low blood perfusion of peripheral nerves in the pathogenesis of DPN deserves more attention. Decreased endoneurial blood flow is observed early in DPN, and oxygen supplementation or treatment with vasodilators has beneficial effects [5]. In addition, hindquarter ischemia and decreased vascular density are found in diabetic animals [6].

Hypoxia-inducible factor-1 (HIF-1) is found in all of the extant metazoan species that have been analyzed [7] and consists of HIF-1*α* and HIF-1*β* subunits [8]. The transcriptional regulator HIF-1*α* is the oxygen regulatory subunit of HIF-1 and the key factor in the HIF-1-mediated response to hypoxia. Under hypoxic conditions, the degradation of HIF-1*α* is weakened, while its transcriptional activity is strengthened [9]. The increased active HIF-1*α* localizes to the nucleus and combines with HIF-1*β*, forming HIF-1, which is able to increase the expression of a series of target genes that improve the adaptability of cells and tissues to low-oxygen conditions [8]. The potent proangiogenic factor vascular endothelial growth factor (VEGF) stimulates the proliferation and migration of endothelial cells and the formation of new blood vessels by increasing vascular permeability and changing the composition of the extracellular matrix. In general, HIF-1*α* and angiogenic growth factors, such as VEGF, are necessary for endothelial progenitor cell (EPC) recruitment to ischemic sites to achieve neovascularization [10].

For thousands of years, natural products for medicine and health have been very important [11], and clinical studies have demonstrated that Chinese herbal medicine (CHM) can be more effective than conventional medicine [12]. In addition, CHM has been approved for use in the treatment of DPN [13]. In China, a large number of clinical trials have demonstrated that the CHM prescription Tangkuei Decoction for Frigid Extremities (TDFE) can relieve the clinical symptoms of DPN. To date, no adverse health effects have been observed due to the clinical application of TDFE.

Clinical methods of diagnosing DPN are not objective or reproducible [14]. Thus, preventive administration of therapeutics deserves more attention. In the present study, the preventive effect of TDFE on DPN was investigated. We hypothesized that TDFE would be able to protect diabetic patients from the effects of DPN. The experiments were divided into two stages (6 weeks and 12 weeks) to evaluate chronic and acute-onset DPN. The plantar thermal stimulation reaction time threshold and nerve conduction velocity were used as neurophysiological measurements, and the endothelial marker von Willebrand factor (vWF) was used to estimate vasa nervorum density. The levels of HIF-1*α* and VEGF in sciatic nerves were also investigated as a first step in exploring the molecular mechanism of action of TDFE.

## 2. Materials and Methods

### 2.1. Animals

Fifty-five 6-week-old male Sprague-Dawley rats weighing 200 ± 20 g were purchased from the Experimental Animal Center of Dalian Medical University. All of the rats were raised at 22 ± 3°C, with 50% humidity and a 12 h light/dark cycle. The rats were randomly divided into 6 groups one week after adaptive feeding: 7 rats in the 6-week blank control (6wBC) group, 10 rats in the 6-week diabetic model (6wDM) group, 9 rats in the 6-week medicine prophylaxis (6wMP) group, 8 rats in the 12-week blank control (12wBC) group, 11 rats in the 12-week diabetic model (12wDM) group, and 10 rats in the 12-week medicine prophylaxis (12wMP) group. During this period, one rat from the 12wBC group was removed from the experiment due to an accidentally injured toe. All of the experimental procedures were approved in advance by the Animal Care and Use Committee of Dalian Medical University and were performed in compliance with the National Institutes of Health Guidelines for the Care and Use of Laboratory Animals.

### 2.2. Materials and Instruments

The herbal components of TDFE, including Radix Angelicae Sinensis (traditional Chinese name (TCN): Danggui), Cassia Twig (TCN: Guizhi), Radix Paeonia Alba (TCN: Baishao),* Asarum sieboldii* Mig (TCN: Xixin), Radix liquiritiae (TCN: Gancao),* Akebia quinata* Decne. (TCN: Mutong), and Fructus Ziziphi Jujubae (TCN: Dazao), were purchased from the Pharmacy Department of the Second Affiliated Hospital of Dalian Medical University. The HIF-1*α* (K377) polyclonal antibody, the VEGF-A polyclonal antibody, and the vWF polyclonal antibody were purchased from Bioworld, Dalian Xinhua Biotechnology Co., Ltd., Dalian, China. The peroxidase-conjugated AffiniPure goat anti-rabbit IgG(H+L) and DAB kit ZSGB-BIO were purchased from Dalian Xinhua Biotechnology Co., Ltd., Dalian, China. The BCA Protein Assay Kit was purchased from Pierce, and the SDS-PAGE kit was purchased from Biotesh, Biotesh Biological & Technology Co., Ltd., Beijing China. RNAiso Plus (9108) and the PrimeScript RT Reagent Kit with gDNA Eraser (RR047A) were purchased from TaKaRa Biotechnology Co., Ltd., Dalian, China. The Thermal-Tingling Apparatus (PL-200) and the biological function experiment system (BL-420F) were purchased from Taimeng, Chengdu Taimeng Software Co., Ltd., Chengdu, China. The BX51 multifunctional microscope was purchased from OLYMPUS Co., Ltd., Beijing, China. The electrophoresis systems (DYCZ 24DN and DYCZ 40D) were purchased from the Beijing Liuyi Instrument Plant, Beijing, China. The TaKaRa PCR Thermal Cycler Dice Real-Time System TP800 was purchased from TaKaRa Biotechnology Co., Ltd., Dalian, China.

### 2.3. Procedures

The four groups (6wDM, 6wMP, 12wDM, and 12wMP) of rats were fasted for 12 hours and then received a single intraperitoneal injection of streptozotocin (50 mg/kg body weight) in citrate buffer at 0.1 mol/L, pH 4.4. After 72 hours, the glucose level of blood collected from the caudal vein was measured, and rats displaying glucose levels greater than 16.7 mmol/L were selected. Blood glucose was monitored once every other day. Rats with higher glucose levels were injected with insulin so that all of the rats had similar blood glucose levels. Establishment of the model failed in one rat in each of the 6wDM, 6wMP, and 12wDM groups. Two rats in the 12wDM group, one in the 6wMP group, and one in the 12wMP group died due to extremely high glucose levels. Three rats (one each from the 6wDM, 12wDM, and 12wMP groups) were removed from the study because their blood glucose levels did not remain elevated during the experiment.

### 2.4. Drug Administration

TDFE was decocted in the Pharmacy Department of the Second Affiliated Hospital of Dalian Medical University. A unified composition proportion was set according to “Fang Ji Xue” (First Edition, 2013.1) published by the China Press of Traditional Chinese Medicine (Radix Angelicae Sinensis : Cassia Twig : Radix Paeonia Alba :* Asarum sieboldii* Mig : Radix liquiritiae :* Akebia quinata* Decne. : Fructus Ziziphi Jujuba = 4 : 3 : 3 : 1 : 2 : 2 : 10). The effective dosage of TDFE in humans is approximately 1.7 g/kg/d. Rats require approximately 5.7 times the amount of a drug as patients, and intragastric administration requires double the amount needed for oral administration. Thus, a concentration of 1.6 g of crude drug in 1 mL of distilled water was used. The TDFE at a dosage of 12 mL/kg/d was administered to the rats in the 6wMP and 12wMP groups, while the other rats were given distilled water at the same dosage. Intragastric administration was sustained for 6 weeks in the 6wBC, 6wDM, and 6wMP groups and for 12 weeks in the 12wBC, 12wDM, and 12wMP groups. Stomach needles were replaced once weekly to protect the rats. One rat in the 6wDM group died due to an error in intragastric administration.

### 2.5. Plantar Testing

By the end of the 6th week, the plantar thermal stimulation reaction time thresholds of the rats in the 6wBC, 6wDM, and 6wMP groups (the 6w rats) were tested. After the apparatus was turned on, it was preheated for one minute. The stop time was set to 30 seconds, and the laser intensity was set to 50%. The rats were placed in the upper boxes until they were motionless. The heat radiation box on the ground floor was moved so that the emission hole was aligned with the center part of the rats' forefeet. The host computer started counting automatically as soon as the launch button was pressed, and timing was discontinued when the rats raised their tested feet. The two rear feet were tested as well, and average results were determined. The thresholds of the rats in the 12wBC, 12wDM, and 12wMP groups (the 12w rats) were measured in the same way at the end of the 12th week.

### 2.6. Electrophysiology

**Figure 1:**
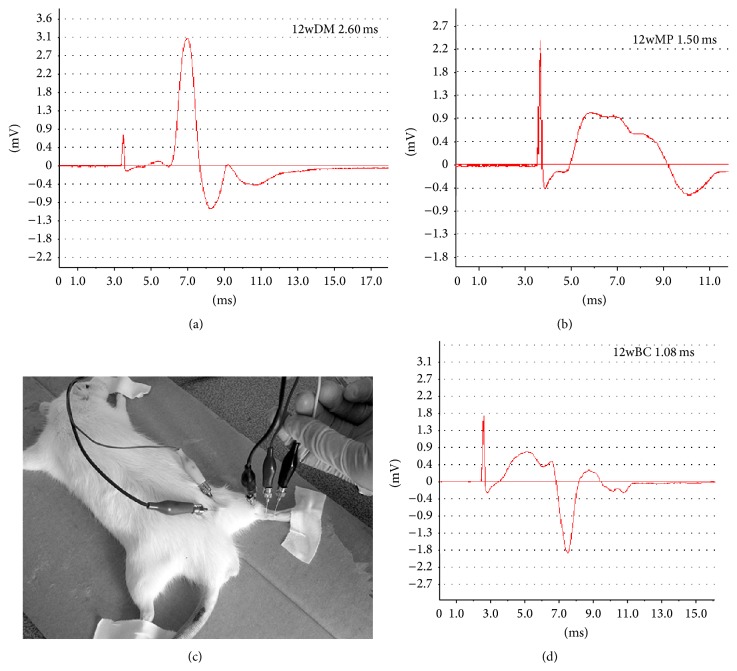
Testing of excitatory signal conduction time in sciatic nerves. (a) Electroneuronography of a rat from the 12-week diabetic model group. (b) Electroneuronography of a rat from the 12-week medicine prophylaxis group. (c) The site of conduction velocity determination. (d) Electroneuronography of a rat from the 12-week blank control group. The abscissa represents the conduction time (ms), and the ordinate represents the electrical potential (mV). ms: millisecond. mV: millivolt.

At the end of the 6th week, the sciatic nerve conduction velocities (SNCVs) of the 6w rats were measured in accordance with published procedures [15]. After the intraperitoneal injection of 10% chloral hydrate (3 mL/kg), the rats were fixed on the operating table in the prone position at a temperature of 20 ± 1°C. The stimulating electrode was placed in the sciatic notch, where the sciatic nerve exits the pelvis, and the recording electrode was placed in the ankle, through which the sciatic nerve passes. The reference electrode was placed between them. A single square wave pulse with a width of 0.1 ms was used. The stimulus intensity, which permitted the presence or absence of an action potential, was varied to determine the stimulus threshold. An intensity of 1.5 times the stimulation threshold was used, and the nerve potential waveform was recorded. The period from the beginning of the stimulation to the occurrence of an action potential was defined as the conduction time of the excitatory signal. The right rear leg of each rat was straightened to create an angle of 45° with the spine. Electroneuronography was performed using a BL-420F biological function experiment system (Figure 1). The distance between the stimulating electrode and the recording electrode was also measured. Then, the SNCV was determined using the formula* SNCV* = distance between the two electrodes/conduction time of the excitatory signal. The measurements were repeated three times and the results were averaged. The 12w rats were tested in the same way at the end of the 12th week.

### 2.7. Immunohistochemistry

Six weeks after the diabetes model was established, 10% chloral hydrate (3 mL/kg) was injected into the 6w rats. The sciatic nerves (approximately 1 cm) were excised, fixed in 4% paraformaldehyde for 24 h, and embedded in paraffin. Subsequently, 3-*μ*m thick sections were prepared. Endogenous peroxidases were inactivated by 3% H_2_O_2_ for 20 min, and the antigen was repaired in CB buffer. The HIF-1*α*, VEGF, and vWF antibodies (1 : 50 dilution) were applied to the sections at 37°C for 1 hour and subsequently at 4°C for one night. The next morning, peroxidase-conjugated AffiniPure goat anti-rabbit IgG was applied at 37°C for 30 minutes. DAB was used for visualization, and the reaction time was controlled under a microscope. After sufficient color had developed, the specimens were dehydrated and sealed. PBS was used as a negative control. Photos were taken using a multifunctional microscope and analyzed with Image-Pro Plus.

### 2.8. Western Blotting

The sciatic nerves were placed in liquid nitrogen as soon as they were extracted from the rats and were dissected, desheathed, and homogenized in ice-cold lysis buffer (1 *μ*g of nerve tissue was added to 10 *μ*L of RIPA) supplemented with protease inhibitors (1 mL of RIPA required 10 *μ*L of PMSF). The homogenates were allowed to stand for 30 minutes and then centrifuged at 10,000 rpm for 15 minutes at 4°C. The supernatants were collected, and the BCA Protein Assay Kit was used to ensure that all of the samples had the same protein concentration. The samples were mixed with loading buffer supernatants and placed in water at 95°C for 10 min. Tissue lysates (32 *μ*g of protein) were subjected to SDS–PAGE (8% acrylamide gels) at 120 V for 55 minutes and then electrotransferred at 250 mA for 120 min onto PVDF membranes. The membranes were blocked with 5% nonfat dry milk for 1 hour and incubated with the HIF-1*α* (K377) polyclonal antibody or the VEGF-A polyclonal antibody at 4°C for one night. After the membranes were washed, they were incubated with peroxidase-conjugated AffiniPure goat anti-rabbit IgG(H+L). The antigen-antibody complexes were visualized by enhanced chemiluminescence detection, and the protein bands were analyzed with Image J2x.

### 2.9. Real-Time PCR

After total RNA was extracted from the sciatic nerves and assessed by electrophoresis, it was used as a template to create cDNA for PCR. The following primers were used: TCTAGTGAACAGGATGGAATGGAG (sense) and TCGTAACTGGTCAGCTGTGGTAA (antisense) for HIF-1*α*; and GGAGATTACTGCCCTGGCTCCTA (sense) and GACTCATCGTACTCCTGCTTGCTG (antisense) for *β*-actin. Using SYBR Green I, the cDNA that was obtained from the reverse transcription of the total RNA (500 ng/*μ*L) extracted from the 6wBC rats was used as a standard to obtain standard curves for HIF-1*α* and *β*-actin. Each sample (100 ng) was compared with the standard curves to determine the relative level of HIF-1*α* mRNA.

### 2.10. Statistical Analysis

SPSS 13.0 was used, and all of the numerical data are expressed as the *x* ± *s*. The significance of differences between groups was analyzed by single-factor analysis of variance. Student's *t*-test was used for comparisons between two groups. The correlation of two variables was analyzed by Pearson's test. Significance was defined at *P* < 0.05.

## 3. Results

### 3.1. Plantar Thermal Stimulation Reaction Time Threshold

As shown in Table 1, the plantar thermal stimulation reaction time thresholds of the rats in the 6wBC, 6wDM, and 6wMP groups did not significantly differ (*P* > 0.05). Compared to the rats in the 12wBC group, the rats in the 12wDM and 12wMP groups exhibited longer plantar thermal stimulation reaction times (*P* < 0.05). In addition, the reaction time of the 12wDM rats was longer than that of the 12wMP rats (*P* < 0.05).

### 3.2. Sciatic Nerve Conduction Velocity

There were no significant differences in sciatic nerve conduction velocity among the rats in the 6wBC, 6wDM, and 6wMP groups (*P* > 0.05). Compared to that of the rats in the 12wBC group, the sciatic nerve conduction velocity of the rats in the 12wDM and 12wMP groups was slower (*P* < 0.05), and the conduction velocity of the 12wDM rats was slower than that of the 12wMP rats (*P* < 0.05). The results are described in Table 2.

### 3.3. HIF-1*α* mRNA, HIF-1*α* Protein, and VEGF Protein Levels

**Figure 2:**
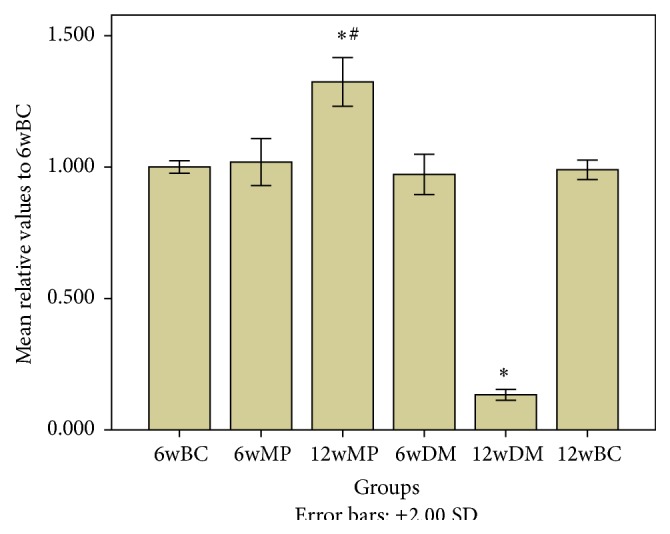
Relative levels of HIF-1*α* mRNA. ^*∗*^ *P* < 0.05 versus the 12wBC group. ^#^ *P* < 0.05 versus the 12wDM group. 6wBC: 6-week blank control group. 6wMP: 6-week medicine prophylaxis group. 12wMP: 12-week medicine prophylaxis group. 6wDM: 6-week diabetic model group. 12wDM: 12-week diabetic model group. 12wBC: 12-week blank control group.

**Figure 3 fig3:**
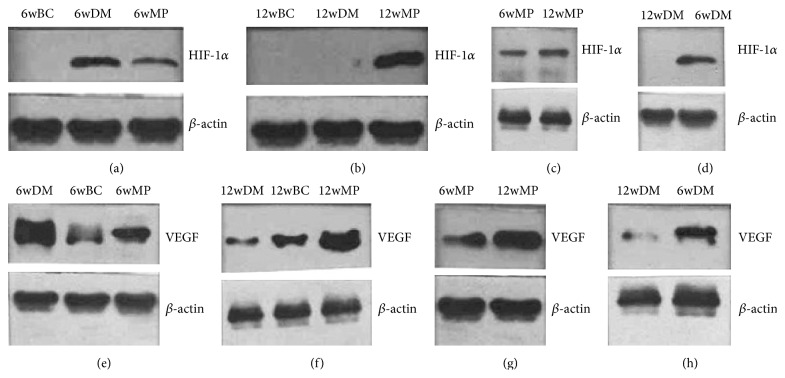
Western blot analysis of protein expression. (a) Comparison of HIF-1*α* protein levels in the 6wBC, 6wDM, and 6wMP groups. (b) Comparison of HIF-1*α* protein levels in the 12wBC, 12wDM, and 12wMP groups. (c) Comparison of HIF-1*α* protein levels between the 6wMP and 12wMP groups. (d) Comparison of HIF-1*α* protein levels between the 12wDM and 6wDM groups. (e) Comparison of VEGF protein levels in the 6wBC, 6wDM, and 6wMP groups. (f) Comparison of VEGF protein levels in the 12wBC, 12wDM, and 12wMP groups. (g) Comparison of VEGF protein levels between the 6wMP and 12wMP groups. (f) Comparison of VEGF protein levels between the 12wDM and 6wDM groups. 6wBC: 6-week blank control group. 6wDM: 6-week diabetic model group. 6wMP: 6-week medicine prophylaxis group. 12wBC: 12-week blank control group. 12wDM: 12-week diabetic model group. 12wMP: 12-week medicine prophylaxis group. *β*-actin protein was used as an internal control.

**Figure 4 fig4:**
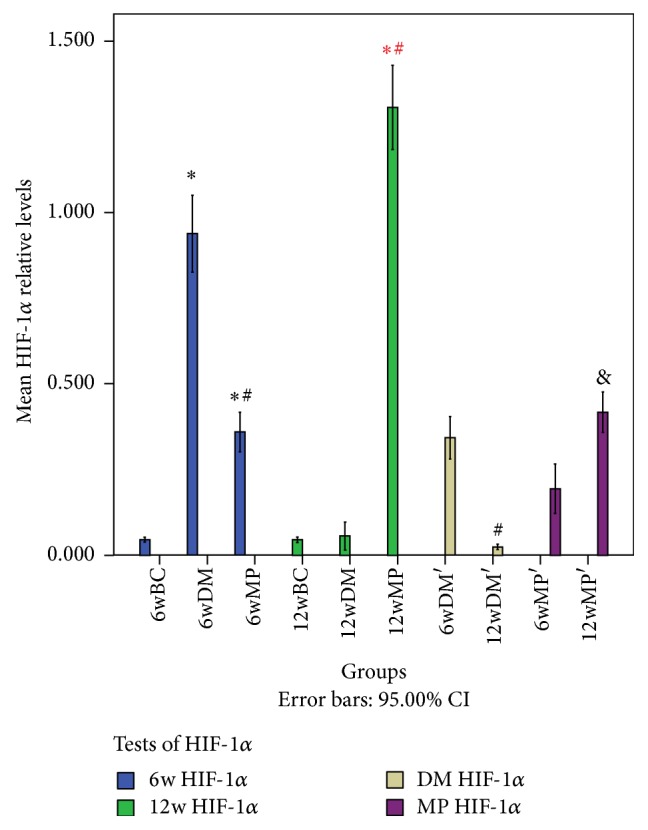
Western blot analysis of HIF-1*α* protein expression. ^*∗*^ *P* < 0.05 versus the 6wBC group. ^#^ *P* < 0.05 versus the 6wDM group. ^*∗*^ *P* < 0.05 versus the 12wBC group. ^#^ *P* < 0.05 versus the 12wDM group. ^&^ *P* < 0.05 versus the 6wMP group. 6w HIF-1*α*: comparison of HIF-1*α* protein levels in the 6wBC, 6wDM, and 6wMP groups. 12w HIF-1*α*: comparison of HIF-1*α* protein levels in the 12wBC, 12wDM, and 12wMP groups. DM HIF-1*α*: comparison of HIF-1*α* protein levels between the 6wDM and 12wDM groups. MP HIF-1*α*: comparison of HIF-1*α* protein levels between the 6wMP and 12wMP groups. 6wBC: 6-week blank control group. 6wDM: 6-week diabetic model group. 6wMP: 6-week medicine prophylaxis group. 12wBC: 12-week blank control group. 12wDM: 12-week diabetic model group. 12wMP: 12-week medicine prophylaxis group.

**Figure 5:**
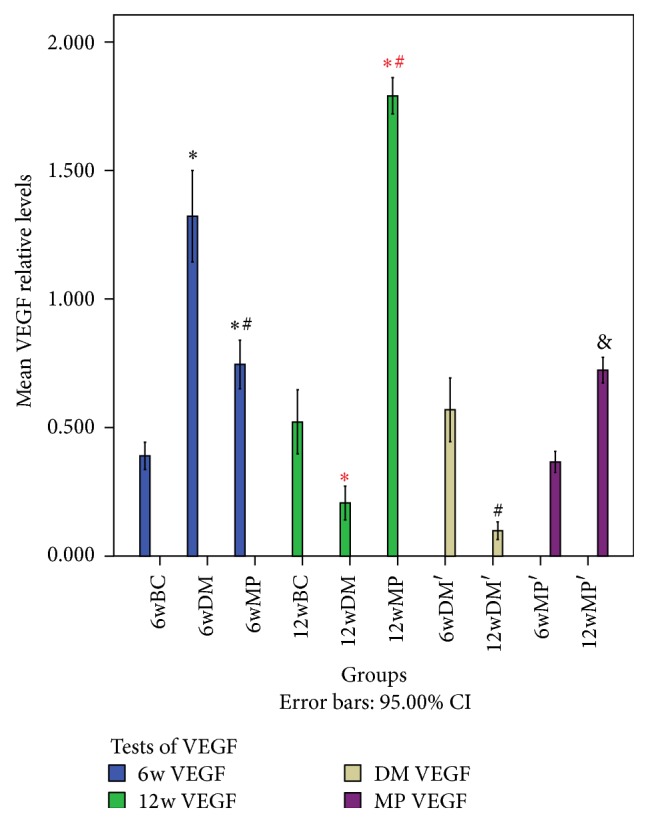
Western blot analysis of VEGF protein expression. ^*∗*^ *P* < 0.05 versus the 6wBC group. ^#^ *P* < 0.05 versus the 6wDM group. ^*∗*^ *P* < 0.05 versus the 12wBC group. ^#^ *P* < 0.05 versus the 12wDM group. ^&^ *P* < 0.05 versus the 6wMP group. 6w VEGF: comparison of VEGF protein levels in the 6wBC, 6wDM, and 6wMP groups. 12w VEGF: comparison of VEGF protein levels in the 12wBC, 12wDM, and 12wMP groups. DM VEGF: comparison of VEGF protein levels between the 6wDM and 12wDM groups. MP VEGF: comparison of VEGF protein levels between the 6wMP and 12wMP groups. 6wBC: 6-week blank control group. 6wDM: 6-week diabetic model group. 6wMP: 6-week medicine prophylaxis group. 12wBC: 12-week blank control group. 12wDM: 12-week diabetic model group. 12wMP: 12-week medicine prophylaxis group.

There were no significant differences in HIF-1*α* mRNA levels among the 6w groups (*P* > 0.05). Compared to the levels in the nerves of the 6wBC groups, HIF-1*α* and VEGF protein levels in the sciatic nerves of the 6wDM and 6wMP groups were higher (*P* < 0.05), while the levels of HIF-1*α* and VEGF protein in the 6wMP sciatic nerves were lower than those in the 6wDM nerves (*P* < 0.05). Compared to those of the 6wDM group, the sciatic nerves of the 12wDM group expressed less HIF-1*α* mRNA, HIF-1*α* protein, and VEGF protein (*P* < 0.05). The 12wMP nerves showed higher levels of HIF-1*α* mRNA, HIF-1*α* protein, and VEGF protein than the 6WMP nerves (*P* < 0.05). There were no significant differences in the levels of HIF-1*α* protein between 12wBC and 12wDM groups (*P* > 0.05). Compared to those of the 12wBC rats, the sciatic nerves of the 12wDM rats expressed less HIF-1*α* mRNA and VEGF protein (*P* < 0.05). In contrast, the sciatic nerves of the 12wMP group expressed significantly more HIF-1*α* mRNA, HIF-1*α* protein, and VEGF protein than those of the 12wBC group (*P* < 0.05). Compared to those in the nerves of the 12wDM rats, the levels of HIF-1*α* mRNA, HIF-1*α* protein, and VEGF protein in the sciatic nerves of the 12wMP rats were significantly higher (*P* < 0.05). The VEGF protein levels in the sciatic nerves depended on the HIF-1*α* protein levels, as reflected by a statistically significant correlation (*r* = 0.945, *P* < 0.05) (Figures 2 [Fig fig3] [Fig fig4]–5).

### 3.4. HIF-1*α*, VEGF, and vWF Expression

**Figure 6:**
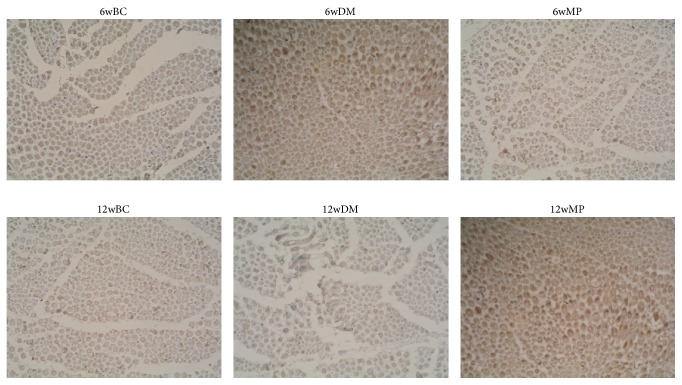
Photomicrographs of VEGF expression in the sciatic nerve tissue. Original magnification: ×400. 6wBC: 6-week blank control group. 6wDM: 6-week diabetic model group. 6wMP: 6-week medicine prophylaxis group. 12wBC: 12-week blank control group. 12wDM: 12-week diabetic model group. 12wMP: 12-week medicine prophylaxis group.

**Figure 7:**
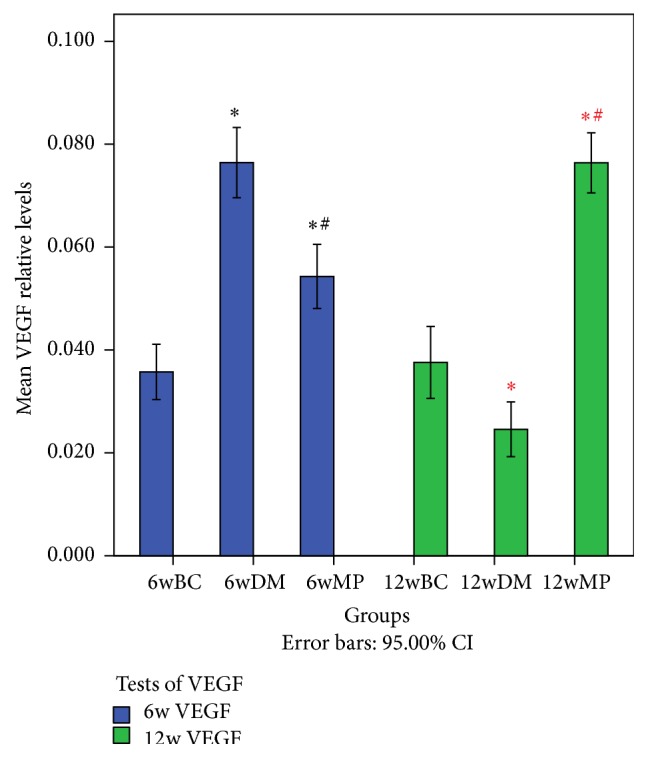
Immunohistochemical analysis of VEGF in the sciatic nerves of the rats. ^**∗**^ *P* < 0.05 versus the 6wBC group. ^#^ *P* < 0.05 versus the 6wDM group. ^*∗*^ *P* < 0.05 versus the 12wBC group. ^#^ *P* < 0.05 versus the 12wDM group. 6w VEGF: comparison of VEGF expression in the sciatic nerves of rats in the 6wBC, 6wDM, and 6wMP groups. 12w VEGF: comparison of VEGF expression in the sciatic nerves of rats in the 12wBC, 12wDM, and 12wMP groups. 6wBC: 6-week blank control group. 6wDM: 6-week diabetic model group. 6wMP: 6-week medicine prophylaxis group. 12wBC: 12-week blank control group. 12wDM: 12-week diabetic model group. 12wMP: 12-week medicine prophylaxis group.

**Figure 8:**
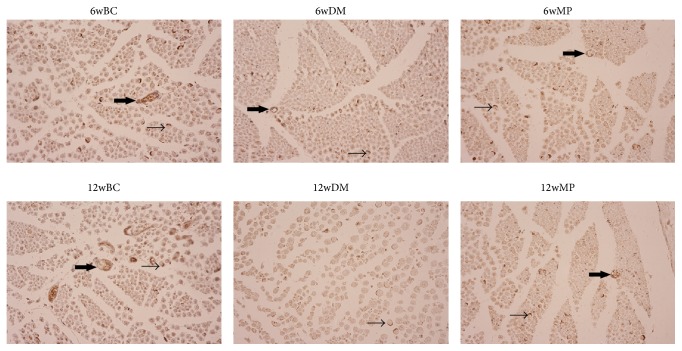
Photomicrographs of vWF expression in the sciatic nerve tissue. Original magnification: ×400. vWF is found on the endoneurial (small arrow) and epineurial (large arrow) vessels. 6wBC: 6-week blank control group. 6wDM: 6-week diabetic model group. 6wMP: 6-week medicine prophylaxis group. 12wBC: 12-week blank control group. 12wDM: 12-week diabetic model group. 12wMP: 12-week medicine prophylaxis group.

**Figure 9:**
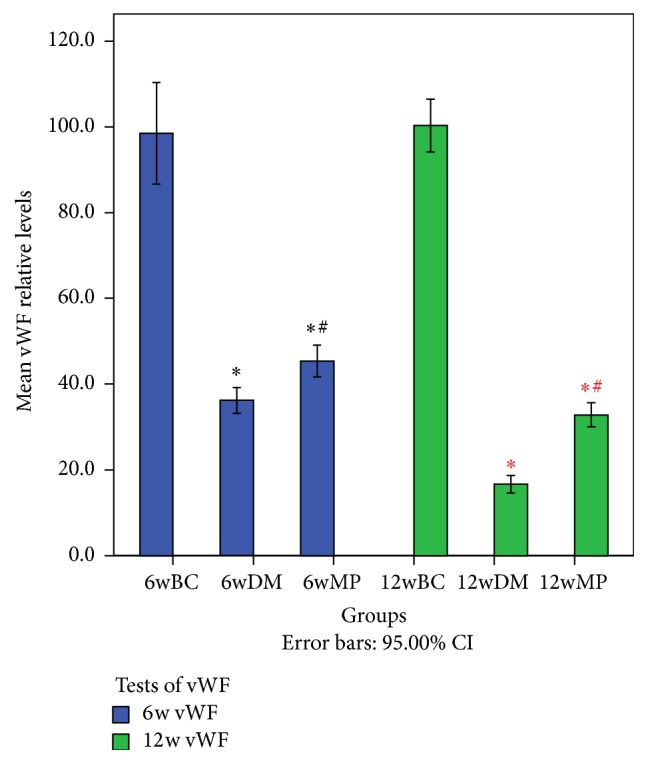
Immunohistochemical analysis of vWF in the sciatic nerves of the rats. ^**∗**^ *P* < 0.05 versus the 6wBC group. ^#^ *P* < 0.05 versus the 6wDM group. ^*∗*^ *P* < 0.05 versus the 12wBC group. ^#^ *P* < 0.05 versus the 12wDM group. 6w vWF: comparison of the vWF expression in the sciatic nerves of rats in the 6wBC, 6wDM, and 6wMP groups. 12w vWF: comparison of the vWF expression among the sciatic nerves of rats from 12wBC, 12wDM, and 12wMP. 6wBC: 6-week blank control group. 6wDM: 6-week diabetic model group. 6wMP: 6-week medicine prophylaxis group. 12wBC: 12-week blank control group. 12wDM: 12-week diabetic model group. 12wMP: 12-week medicine prophylaxis group.

HIF-1*α*-positive cells were not found in all of the groups. VEGF expression was found in small and medium neurons as well as in the membranes, axons, and myelin sheath of the sciatic nerves. As shown in Figures 6 and 7, the expression of VEGF in the 6wDM and 6wMP groups was higher than that in the 6wBC group (*P* < 0.05), and more VEGF was expressed in the 6wDM group than in the 6wMP group (*P* < 0.05). The expression of VEGF in the 12wDM group was lower than that in the 12wBC group (*P* < 0.05), while more VEGF was expressed in the 12wMP group than in the 12wBC group (*P* < 0.05). vWF immunoreactivity was detected in endoneurial and epineurial vessels. As shown in Figures 8 and 9, the expression of vWF in the 6wDM and 6wMP groups was lower than that in the 6wBC group (*P* < 0.05), and less vWF was expressed in the 6wDM group than in the 6wMP group (*P* < 0.05). The expression of vWF in the 12wDM and 12wMP groups was lower than that in the 12wBC group (*P* < 0.05), while more vWF was expressed in the 12wMP group than in the 12wDM group (*P* < 0.05).

## 4. Discussion

The present study clearly demonstrated that TDFE relieved the decrease in plantar thermal sensation and sciatic nerve conduction velocity as well as vasa nervorum injury in diabetic rats. TDFE delayed the development of DPN, and its preventive function was closely associated with HIF-1*α* and VEGF. No adverse health effects of TDFE administration were observed throughout the course of the experiments.

The HIF-1*α* subunit is virtually undetectable under normoxic conditions due to its very short half-life of less than 5 min; it is rapidly degraded by the ubiquitin-proteasome pathway [16]. Because immunohistochemistry was performed under normoxic conditions, cells expressing HIF-1*α* were not found in all of the groups. vWF is produced in endothelium, megakaryocytes and subendothelial connective tissue [17]. It was used as an endothelial marker to identify endoneurial and epineurial vessels in sciatic nerves. The low expression of vWF in the 6wDM rats and 12wDM rats indicates that the vasa nervorum of the rats were damaged by the diabetes. The increased expression of vWF in the 6wMP rats and 12wMP rats suggests that the application of TDFE relieved the ischemia and hypoxia of the sciatic nerves. The ischemia-induced production of growth factors (including VEGF) has been clearly shown to be decreased in diabetic tissues and in hyperglycemia [18, 19]. Similar immunohistochemistry results were obtained for the sciatic nerves of the 12wDM rats.

Nerve regeneration requires the coordination of multiple cell types. According to Cattin et al. [20], macrophages are sensitive to hypoxia in peripheral nerves. Upon hypoxia, the transcription factor HIF-1*α* is stabilized and initiates a transcriptional response that induces angiogenesis by upregulating VEGF. The increased expression of VEGF in macrophages stimulates EC proliferation and migration, resulting in the formation of new sciatic nerve tissue.

At 6 weeks, the damage to the sciatic nerves was slight; thus, the rats showed no significant differences in thermal sensation or sciatic nerve conduction velocity. However, the sciatic nerves of the diabetic rats suffered ischemia and hypoxia, and VEGF was adaptively upregulated in a manner that depended on activated HIF-1*α*. Thus, compared to those of the 6wBC group, the sciatic nerves of the diabetic rats expressed more HIF-1*α* and VEGF protein; however, this upregulation was transient. The 12wDM rats exhibited very low levels of HIF-1*α* protein and less VEGF protein than the 6wDM rats due to the insufficient protective effect of activated HIF-1*α* against long-term hypoxia; this deficiency leads to apoptosis, which may have contributed to the abnormal thermal sensation and sciatic nerve conduction velocity [5]. Comparisons between the 6wMP and 6wDM groups and between the 6wMP and 12wMP groups revealed that the HIF-1*α* and VEGF protein levels in the sciatic nerves of the 6wMP rats were lower than those in the 6wDM rats and were in a rising stage. The sciatic nerves of the 6wMP rats experienced less severe ischemia and hypoxia than the 6wDM rats, which we attribute to the effects of TDFE.

By 12 weeks, the sciatic nerves of the diabetic rats were obviously injured, as can easily occur with the development of diabetes. The nerves of the 12wDM group were more severely damaged than those of the 12wMP group. A reduction in HIF-1*α* protein decreases the expression of genes that are activated by HIF-1, as demonstrated by the expression of VEGF in the 12wDM rats. Low VEGF expression is closely related to DPN [21] and is one of the reasons for the differences in thermal sensation and nerve conduction velocity among the 12w rats. In contrast, the 12wMP rats exhibited high levels of HIF-1*α* and VEGF protein, which protected the sciatic nerves against diabetes-induced degeneration. The enhanced expression of VEGF improved the reduced thermal sensation, the decrease in nerve conduction velocity, and the obstacle of vascular regeneration [22]. This result supports our hypothesis.

The results from the 12w rats and 6w rats are not contradictory. During the early stages of diabetes, HIF-1*α* and VEGF are signals that respond to the degree of tissue ischemia and hypoxia. Lower levels of HIF-1*α* and VEGF indicate milder ischemia and hypoxia of the sciatic nerves. However, when the disease is advanced, the body is no longer able to compensate. Decreased VEGF leads to decreased function and recruitment of circulating angiogenic cells, injured sciatic nerves, and the poor growth, proliferation, and adhesion of endothelial cells [23, 24]. HIF-1*α* and VEGF become manifestations of the ability to resist ischemia/hypoxia and to form new blood vessels. Increased levels of HIF-1*α* and VEGF lessen the severity of ischemia and hypoxia of the sciatic nerves. In terms of protein expression levels, the results obtained in the 6w and 12w rats led us to the same conclusion: TDFE was able to relieve the ischemia and hypoxia of the sciatic nerves, suggesting that TDFE can prevent the development of DPN, reducing injury to the sciatic nerves in diabetic rats.

HIF-1*α* was regulated at the post-mRNA level during the first six weeks. However, the expression of HIF-1*α* mRNA differed between the groups by 12 weeks. Due to long-term low blood perfusion and the low-oxygen environment, the partial oxygen pressure of the sciatic nerve remained low, with a small degree of variation. This further damaged the sensitivity of the sciatic nerves to hypoxia. Thus, the HIF-1*α* mRNA levels in the sciatic nerves of the 12wDM rats were even lower than those in the 12wBC rats. Surprisingly, the highest HIF-1*α* mRNA levels occurred in the sciatic nerves of the 12wMP rats. Many studies [25–27] have indicated that the hypoxia-induced increase in HIF-1*α* occurs at the protein level, with no change in the mRNA level. Thus, this study suggests that the upregulation of HIF-1*α* mRNA in the sciatic nerves of the 12wMP rats was very likely to be related to the application of TDFE. We can also conclude that TDFE can increase HIF-1*α* mRNA levels. Increased HIF-1*α* mRNA provides greater protection for sciatic nerves by increasing the levels of HIF-1*α* and VEGF protein.

According to Traditional Chinese Medicine (TCM) theory, long-term diabetes results in deficiencies of Yin, Yang, Qi, and Xue, causing blood stasis and the blockage of meridians (alterations in the blood circulation and afflux to organs and tissues), which plays an important role in the etiology and pathogenesis of DPN [28]. Zhang Zhongjing from the Han Dynasty discussed TDFE in his work called “Treatise on Exogenous Febrile Disease,” in which he suggested that TDFE affected the warming and dredging meridians (passages through which vital energy circulates). This effect is similar to what we would now describe as increasing blood flow. Zhang Zhongjing maintained that “Algid extremities, faint pulses, and Tangkuei Decoction for Frigid Extremities are needed.” Xu Hong from the Ming Dynasty proposed another explanation that “deficiencies of Yin and Xue result in feeble pulses and deficiencies of Yang and Qi cause algid extremities. Angelicae Sinensis (TCN: Danggui) is used to enrich the Xue. Radix Paeonia Alba (TCN: Baishao) is used to nourish Ying Qi. Cassia Twig (TCN: Guizhi) and* Asarum sieboldii* Mig (TCN: Xixin) are used to disperse cold. Radix liquiritiae (TCN: Gancao),* Akebia quinata* Decne. (TCN: Mutong), and Fructus Ziziphi Jujubae (TCN: Dazao) were used as assist medicine.” Thus, the effects of TDFE fit well with the TCM theory of the pathogenesis of DPN. TDFE can enrich Yin and Xue, invigorate meridians, facilitate Yang and Qi, warm extremities, and recover fine pulses.

TDFE is a classic TCM prescription; it has been used for warming and dredging meridians for more than one thousand years, and its efficacy is unquestionable. In the present study, early preventive administration of TDFE relieved the ischemia and hypoxia of sciatic nerves and maintained thermal sensation and nerve conduction velocity. As the DPN progressed, this preventive effect became increasingly obvious. In addition, the application of TDFE for 12 weeks could protect the sciatic nerves of the diabetic rats by increasing the expression of HIF-1*α* mRNA. However, analysis of the chemical composition of TDFE and studies of other peripheral nerves are still required. In addition, the specific mechanism by which TDFE regulates the levels of HIF-1*α* mRNA remains unclear.

## Figures and Tables

**Table 1:** Plantar thermal stimulation reaction time thresholds of the rats.

Groups	6wBC	6wDM	6wMP	12wBC	12wDM	12wMP
Thresholds (s)	10.78 ± 2.83	12.53 ± 2.74	11.50 ± 2.50	10.74 ± 1.54	25.19 ± 1.82^*∗*^	17.01 ± 3.20^*∗*#^

^*∗*^ *P* < 0.05 versus the 12wBC group. ^#^ *P* < 0.05 versus the 12wDM group. 6wBC: 6-week blank control group. 6wDM: 6-week diabetic model group. 6wMP: 6-week medicine prophylaxis group. 12wBC: 12-week blank control group. 12wDM: 12-week diabetic model group. 12wMP: 12-week medicine prophylaxis group. (s): seconds.

**Table 2:** Sciatic nerve conduction velocities (SNCVs) of the rats.

Groups	6wBC	6wDM	6wMP	12wBC	12wDM	12wMP
SNCV (m/s)	48.03 ± 4.96	44.37 ± 5.17	46.33 ± 3.98	48.68 ± 5.63	29.69 ± 3.31^*∗*^	38.66 ± 3.94^*∗*#^

^*∗*^ *P* < 0.05 versus the 12wBC group. ^#^ *P* < 0.05 versus the 12wDM group. 6wBC: 6-week blank control group. 6wDM: 6-week diabetic model group. 6wMP: 6-week medicine prophylaxis group. 12wBC: 12-week blank control group. 12wDM: 12-week diabetic model group. 12wMP: 12-week medicine prophylaxis group. (m/s): meters/second.

## References

[1] Peltier A., Goutman S. A., Callaghan B. C. (2014). Painful diabetic neuropathy. *British Medical Journal*.

[2] Chen W., Zhang Y., Li X., Yang G., Liu J. P. (2013). Chinese herbal medicine for diabetic peripheral neuropathy. *Cochrane Database of Systematic Reviews*.

[3] Guo L., Jiang F., Tang Y.-T., Si M.-Y., Jiao X.-Y. (2014). The association of serum vascular endothelial growth factor and ferritin in diabetic microvascular disease. *Diabetes Technology and Therapeutics*.

[4] Zheng C., Ou W., Shen H., Zhou Z., Wang J. (2015). Combined therapy of diabetic peripheral neuropathy with breviscapine and mecobalamin: a systematic review and a meta-analysis of Chinese studies. *BioMed Research International*.

[5] Chavez J. C., Almhanna K., Berti-Mattera L. N. (2005). Transient expression of hypoxia-inducible factor-1 alpha and target genes in peripheral nerves from diabetic rats. *Neuroscience Letters*.

[6] Sarkar K., Fox-Talbot K., Steenbergen C., Bosch-Marcë M., Semenza G. L. (2009). Adenoviral transfer of HIF-1*α* enhances vascular responses to critical limb ischemia in diabetic mice. *Proceedings of the National Academy of Sciences of the United States of America*.

[7] Loenarz C., Coleman M. L., Boleininger A. (2011). The hypoxia-inducible transcription factor pathway regulates oxygen sensing in the simplest animal, Trichoplax adhaerens. *The EMBO Reports*.

[8] Semenza G. L. (2012). Hypoxia-inducible factors in physiology and medicine. *Cell*.

[9] Xin X., Rodrigues M., Umapathi M. (2013). Hypoxic retinal Müller cells promote vascular permeability by HIF-1-dependent up-regulation of angiopoietin-like 4. *Proceedings of the National Academy of Sciences of the United States of America*.

[10] Wang C., Cai Y., Zhang Y., Xiong Z., Li G., Cui L. (2014). Local injection of deferoxamine improves neovascularization in ischemic diabetic random flap by increasing HIF-1*α* and VEGF expression. *PLoS ONE*.

[11] Ji H.-F., Li X.-J., Zhang H.-Y. (2009). Natural products and drug discovery: can thousands of years of ancient medical knowledge lead us to new and powerful drug combinations in the fight against cancer and dementia?. *EMBO Reports*.

[12] Chen W., Luo Y.-F., Liu J.-P. (2011). Topical herbal medicine for treatment of diabetic peripheral neuropathy: a systematic review of randomized controlled trials. *Forschende Komplementärmedizin*.

[13] Hao C.-Z., Wu F., Lu L. (2013). Chinese herbal medicine for diabetic peripheral neuropathy: an updated meta-analysis of 10 high-quality randomized controlled studies. *PLoS ONE*.

[14] Selvarajah D., Cash T., Davies J. (2015). SUDOSCAN: a simple, rapid, and objective method with potential for screening for diabetic peripheral neuropathy. *PLoS ONE*.

[15] Yao H.-P., Feng W.-Y., Wei Y.-X. (2011). Methodology of the determination of sciatic nerve conduction velocity in rats. *China Pharmacy*.

[16] Bento C. F., Pereira P. (2011). Regulation of hypoxia-inducible factor 1 and the loss of the cellular response to hypoxia in diabetes. *Diabetologia*.

[17] Sadler J. E. (1998). Biochemistry and genetics of von Willebrand factor. *Annual Review of Biochemistry*.

[18] Gallagher K. A., Liu Z.-J., Xiao M. (2007). Diabetic impairments in NO-mediated endothelial progenitor cell mobilization and homing are reversed by hyperoxia and SDF-1*α*. *Journal of Clinical Investigation*.

[19] Ceradini D. J., Yao D., Grogan R. H. (2008). Decreasing intracellular superoxide corrects defective ischemia-induced new vessel formation in diabetic mice. *The Journal of Biological Chemistry*.

[20] Cattin A., Burden J., Van Emmenis L. (2015). Macrophage-induced blood vessels guide schwann cell-mediated regeneration of peripheral nerves. *Cell*.

[21] Wirostko B., Wong T. Y., Simó R. (2008). Vascular endothelial growth factor and diabetic complications. *Progress in Retinal and Eye Research*.

[22] Schratzberger P., Walter D. H., Rittig K. (2001). Reversal of experimental diabetic neuropathy by VEGF gene transfer. *Journal of Clinical Investigation*.

[23] Fadini G. P., Sartore S., Schiavon M. (2006). Diabetes impairs progenitor cell mobilisation after hindlimb ischaemia-reperfusion injury in rats. *Diabetologia*.

[24] Liu L., Marti G. P., Wei X. (2008). Age-dependent impairment of HIF-1*α* expression in diabetic mice: correction with electroporation-facilitated gene therapy increases wound healing, angiogenesis, and circulating angiogenic cells. *Journal of Cellular Physiology*.

[25] Moritz W., Meier F., Stroka D. M. (2002). Apoptosis in hypoxic human pancreatic islets correlates with HIF-1alpha expression. *The FASEB Journal*.

[26] Wenger R. H., Kvietikova I., Rolfs A., Gassmann M., Marti H. H. (1997). Hypoxia-inducible factor-1*α* is regulated at the post-mRNA level. *Kidney International*.

[27] Huang L. E., Arany Z., Livingston D. M., Franklin Bunn H. (1996). Activation of hypoxia-inducible transcription factor depends primarily upon redox-sensitive stabilization of its *α* subunit. *Journal of Biological Chemistry*.

[28] Piao Y., Liang X. (2012). Chinese medicine in diabetic peripheral neuropathy: experimental research on nerve repair and regeneration. *Evidence-Based Complementary and Alternative Medicine*.

